# Vitamin C and Metabolic Syndrome: A Meta-Analysis of Observational Studies

**DOI:** 10.3389/fnut.2021.728880

**Published:** 2021-10-08

**Authors:** Hongbin Guo, Jun Ding, Qi Liu, Yusheng Li, Jieyu Liang, Yi Zhang

**Affiliations:** ^1^Department of Orthopedics, Xiangya Hospital, Central South University, Changsha, China; ^2^Changsha Social Work College, Changsha, China

**Keywords:** dietary vitamin C, circulating vitamin C, metabolic syndrome, meta-analysis, observational studies

## Abstract

**Background:** The association between vitamin C and metabolic syndrome (MetS) has been evaluated in several epidemiological studies with conflicting results. This meta-analysis was therefore employed to further investigate the above issue.

**Methods:** The observational studies on the associations of dietary and circulating (serum and plasma) vitamin C levels with MetS were searched in the PubMed, Web of Science, and Embase database up to April 2021. The pooled relative risk (RR) of MetS for the highest vs. lowest dietary and circulating vitamin C levels and the standard mean difference (SMD) of dietary and circulating vitamin C levels for MetS vs. control subjects were calculated, respectively.

**Results:** A total of 28 observational studies were identified in this meta-analysis. Specifically, 23 studies were related to the dietary vitamin C level. The overall multivariable-adjusted RR demonstrated that the dietary vitamin C level was inversely associated with MetS (RR = 0.93, 95% CI: 0.88–0.97; *P* = 0.003). Moreover, the overall combined SMD showed that the dietary vitamin C level in MetS was lower than that in control subjects (SMD = −0.04, 95% CI: −0.08 to −0.01; *P* = 0.024). With regard to the circulating vitamin C level, 11 studies were included. The overall multivariable-adjusted RR demonstrated that the circulating vitamin C level was inversely associated with MetS (RR = 0.60, 95% CI: 0.49–0.74; *P* < 0.001). In addition, the overall combined SMD showed that the circulating vitamin C level in MetS was lower than that in control subjects (SMD=-0.82, 95%CI: −1.24 to −0.40; *P* < 0.001).

**Conclusions:** Current evidence suggests that both dietary and circulating vitamin C level is inversely associated with MetS. However, due to the limitation of the available evidence, more well-designed prospective studies are still needed.

## Introduction

Metabolic syndrome (MetS) is defined as the presence of the following five metabolic abnormalities (at least three): elevated waist circumference, blood pressure, fasting blood glucose, triglycerides, and decreased high-density lipoprotein cholesterol (1). Affecting 25% of the population in the developed world, MetS has been considered an important public health issue in parallel to obesity and diabetes (2). Although the etiology of MetS is not well-understood yet, dietary factors are thought to be involved in MetS (3).

Vitamin C, an essential water-soluble micronutrient traditionally utilized to prevent and treat scurvy (also known as ascorbic acid), is one of the most common antioxidants. Fruit and vegetable consumption, which are equipped with abundant vitamin C, have been demonstrated to be inversely associated with MetS in our previous study (4). Moreover, vitamin C consumption is associated with a lower risk of type 2 diabetes (5) and hypertension (6). Fundamentally, the induction of vitamin C deficiency could lead to a phenotype characterized by insulin resistance, weight gain, dyslipidemia, and hepatic steatosis (7). Above all, it is speculated that vitamin C levels may be negatively associated with MetS.

To the best of our knowledge, a number of observational studies have examined the associations of dietary and circulating vitamin C level with MetS (8–35). However, no final conclusion can be obtained. Thus, the present meta-analysis of observational studies was employed to investigate the issue further. It was hypothesized that the dietary and circulating vitamin C was inversely associated with MetS.

## Materials and Methods

### Search Strategy

Our meta-analysis was performed according to the preferred reporting items for systematic reviews and meta-analyses (PRISMA) guidelines (36). The PubMed, Web of Science, and Embase electronic database were searched during April 2021 by using a combination of keywords and in-text words related to MetS (“metabolic syndrome”) and vitamin C (“vitamin C” and “ascorbic acid”). No language and MetS diagnostic criteria restrictions were set in the search strategy. The titles and abstracts of all articles were first screened. Then, the full articles were read to identify the eligible studies. Moreover, the reference lists for the retrieved articles were reviewed to include additional studies.

### Study Selection

Two researchers (YZ and JD) reviewed the titles, abstracts, and full texts of all retrieved studies independently. Disagreements were resolved by discussions and mutual consultations. The included studies were required to meet the following criteria: (1) the study design was an observational study; (2) the outcomes included the associations of dietary and circulating vitamin C level with MetS; and (3) the relative risk (RR), odds ratio (OR), or standard mean difference (SMD) with 95% confidence interval (CI) were reported. The exclusion criteria were listed as follows: (1) duplicated or irrelevant articles; (2) reviews, letters, or case reports; (3) randomized controlled trials; and (4) non-human studies.

### Data Extraction

Two researchers extracted the data (YZ and JD) independently, and disagreements were resolved by consensus. The information about the first author, year of publication, location, age, gender, sample size, study design, adjustments, exposure, category of exposure, effect estimates, adjustments, and diagnostic criteria of MetS, were collected. The corresponding effect estimates with 95% CIs for the highest vs. lowest dietary levels and the circulating vitamin C level were extracted (adjusted for the maximum number of confounding variables). Moreover, the dietary and the circulating vitamin C levels (mean ± SD) were also extracted to calculate the SMD (MetS vs. control).

### Quality Assessment

Quality assessment was conducted according to the Newcastle–Ottawa (NOS) criteria for non-randomized studies, which is based on three broad perspectives: the selection process of study cohorts, the comparability among different cohorts, and the identification of exposure or outcome of study cohorts. Disagreements with respect to the methodological quality were resolved by discussion and mutual consultation. Studies in other languages were translated into English for evaluation. A study awarded seven or more stars were considered high-quality (37), which was the basis for subgroup analysis for study quality.

### Statistical Analyses

The RR for MetS and SMD for dietary and circulating vitamin C levels were the outcome measures in the present study. The *I*^2^ statistic, which measures the percentage of total variation across studies due to heterogeneity, was examined (*I*^2^ > 50% was considered heterogeneity). If significant heterogeneity was observed among the studies, the random-effects model was used; otherwise, the fixed effects model was accepted. Begg's test was employed to assess the publication bias (38). A *p* < 0.05 was considered statistically significant. Moreover, subgroup analysis for study design, diagnostic criteria of MetS, geographical region, sample size, exposure assessment, study quality, adjustment of BMI, and physical activity was conducted for RR analysis. In addition, subgroup analysis for diagnostic criteria of MetS, geographical region, sample size, study quality, and exposure assessment was employed for SMD analysis. Of note, Kim et al. separated their effect estimates by high and low physical activity (PA), which served as two independent datasets (22).

## Results

### Study Identification and Selection

**Figure 1:**
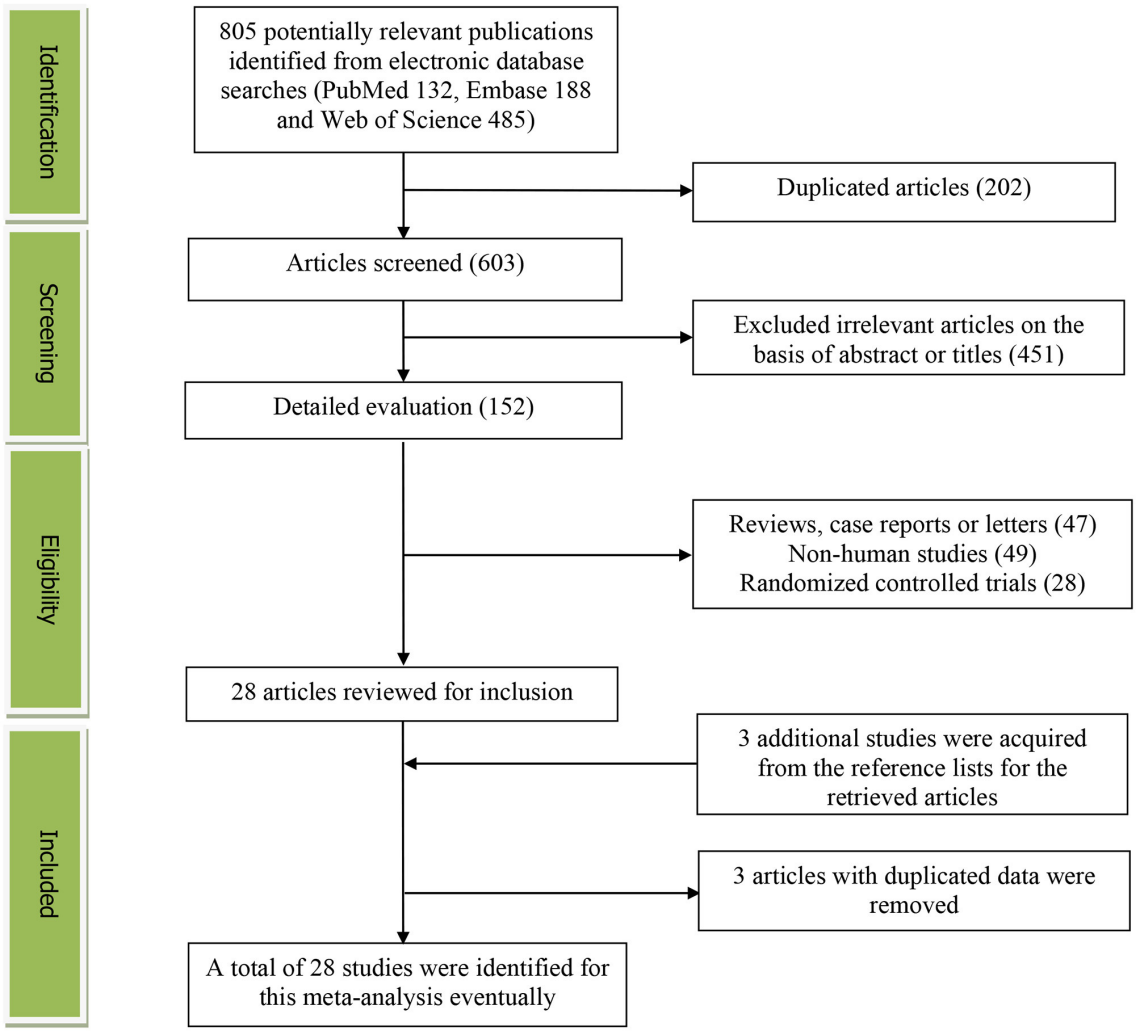
The detailed flow diagram of the study identification and selection in this meta-analysis.

Figure 1 presents the detailed flow diagram of the study identification and selection. A total of 805 potentially relevant articles (PubMed: 132, Embase: 188, and Web of Science: 485) were retrieved during the initial literature search. After eliminating 202 duplicated articles, 603 articles were screened according to the titles and abstracts and 451 irrelevant studies were excluded. Then, 47 reviews, case reports, or letters; 49 non-human studies; and 28 randomized control trials studies were removed, respectively. Thereafter, three additional studies were acquired from the reference lists for the retrieved articles (33–35). Moreover, three studies were excluded for duplicated data (39–41). Eventually, a total of 28 studies were selected for this meta-analysis.

### Study Characteristics

The main characteristics of the included studies are shown in Supplementary Table 1. These studies were published between 2003 and 2021. Sixteen of them were performed in Asian countries [Korea (9–11, 19, 22–25, 35), China (17, 18, 20, 27), Thailand (31), Iran (33), and Saudi Arabia (16)], and four were conducted in European countries [Poland (21, 26), France (29), and Finland (13)]. The other eight studies were from the US (8, 12, 14, 15), Canada (32), Ecuador (28), Brazil (34), and Nigeria (30). Both male and female participants were considered, except for Bruscate's study (34). The sample size ranged from 143 to 27,656 for a total number of 110,771. The dietary vitamin C level was assessed by food-frequency questionnaire (FFQ) in four studies (8, 10, 15, 20), a 24-h or 3-day recall in 18 studies (9, 11, 12, 14, 16–19, 21–27, 33–35), and a 4-day record in one study (13). The criteria for MetS were National Cholesterol Education Program-Adult Treatment Panel III (NCEP ATP III) and International Diabetes Federation (IDF) in 17 (8–13, 17–19, 23–25, 27, 29–31, 35) and seven studies (14, 16, 21, 26, 28, 33, 34), respectively. Moreover, American Heart Association (AHA) (15, 20) and Joint Interim Statement (JIS) (22, 32) were also utilized.

### RR of MetS for the Highest vs. Lowest Dietary Vitamin C Category

**Figure 2:**
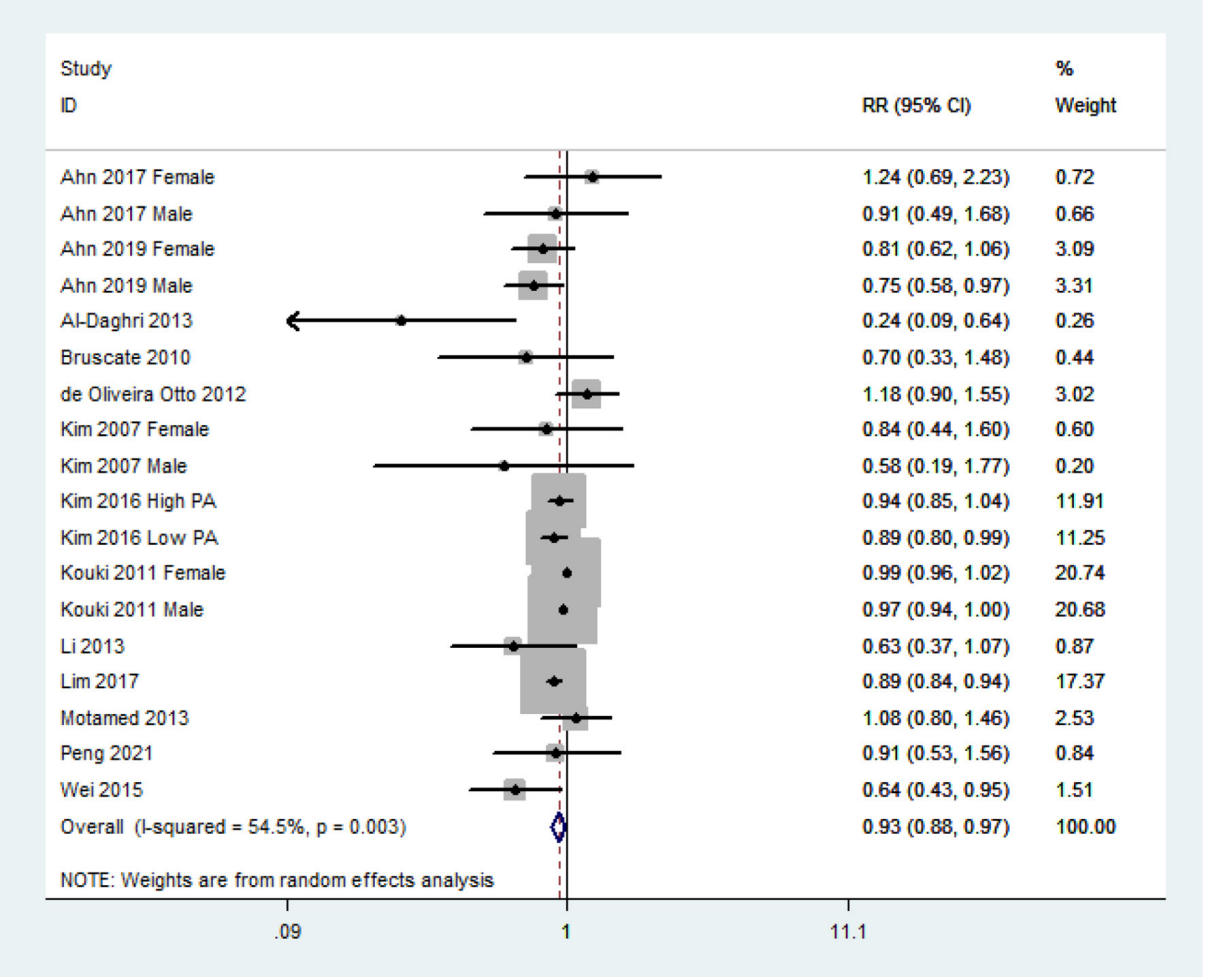
Forest plot of meta-analysis. Overall multi-variable adjusted RR of MetS for the highest vs. lowest category of dietary vitamin C level.

The overall multivariable-adjusted RR demonstrated that the dietary vitamin C level was negatively associated with MetS (RR = 0.93, 95% CI: 0.88–0.97; *P* = 0.003) (Figure 2). A substantial level of heterogeneity was obtained among various studies (*P* = 0.003, *I*^2^ = 54.5%). No evidence of publication bias existed according to Begg's rank correlation test (*P* = 0.495). The results of subgroup analysis are presented in Table 1. The above findings were confirmed in crosssectional (RR = 0.92, 95% CI: 0.87–0.97; *P* = 0.001), NCEP ATP III (RR = 0.94, 95% CI: 0.89–0.99; *P* = 0.01), and 24-h or 3-day recall (RR = 0.89, 95% CI: 0.86 to 0.93; *P* < 0.001) studies.

**Table 1:** Subgroup analysis of MetS for the highest vs. lowest dietary vitamin C level category.

**Stratification**	**Number of studies**	**Pooled RR**	**95% CI**	***P*-value**	**Heterogeneity**
All studies	13	0.93	0.88, 0.97	*P =* 0.003	*P =* 0.003; *I*^2^ = 54%
**Study design**					
Crosssectional	11	0.92	0.87, 0.97	*P =* 0.001	*P =* 0.002; *I*^2^ = 57%
Cohort	2	1.12	0.88, 1.43	*P =* 0.36	*P =* 0.40; *I*^2^ = 0%
**Diagnostic criteria of MetS**					
NCEP ATP III	7	0.94	0.89, 0.99	*P =* 0.01	*P =* 0.03; *I*^2^ = 50%
Other	6	0.91	0.78, 1.05	*P =* 0.19	*P =* 0.01; *I*^2^ = 62%
**Geographical region**					
Asia	9	0.89	0.85, 0.92	*P < * 0.001	*P =* 0.18; *I*^2^ = 26%
Non-Asia	4	0.98	0.96, 1.00	*P =* 0.09	*P =* 0.44; *I*^2^ = 0%
**Sample size**					
<1,000	7	0.88	0.84, 0.93	*P < * 0.001	*P =* 0.22; *I*^2^ = 25%
>1,000	6	0.95	0.91, 1.00	*P =* 0.04	*P =* 0.03; *I*^2^ = 52%
**Exposure assessment**					
FFQ	2	0.88	0.49, 1.61	*P =* 0.69	*P =* 0.01; *I*^2^ = 84%
24 h or 3 days recall	10	0.89	0.86, 0.93	*P < * 0.001	*P =* 0.27; *I*^2^ = 17%
**Study quality**					
High-quality	10	0.94	0.89, 0.99	*P =* 0.02	*P =* 0.01; *I*^2^ = 52%
Low-quality	3	0.89	0.84, 0.94	*P < * 0.001	*P =* 0.82; *I*^2^ = 0%
**Adjustment of BMI**					
Adjusted	6	0.91	0.85, 0.97	*P =* 0.004	*P =* 0.04; *I*^2^ = 50%
Unadjusted	7	0.94	0.90, 0.99	*P* = 0.03	*P =* 0.02; *I*^2^ = 56%
**Adjustment of physical activity**					
Adjusted	8	0.90	0.80, 1.02	*P =* 0.09	*P =* 0.07; *I*^2^ = 41%
Unadjusted	5	0.94	0.89, 0.98	*P =* 0.007	*P =* 0.004; *I*^2^ = 69%

### SMD of the Dietary Vitamin C Level for MetS vs. Control Subjects

**Figure 3:**
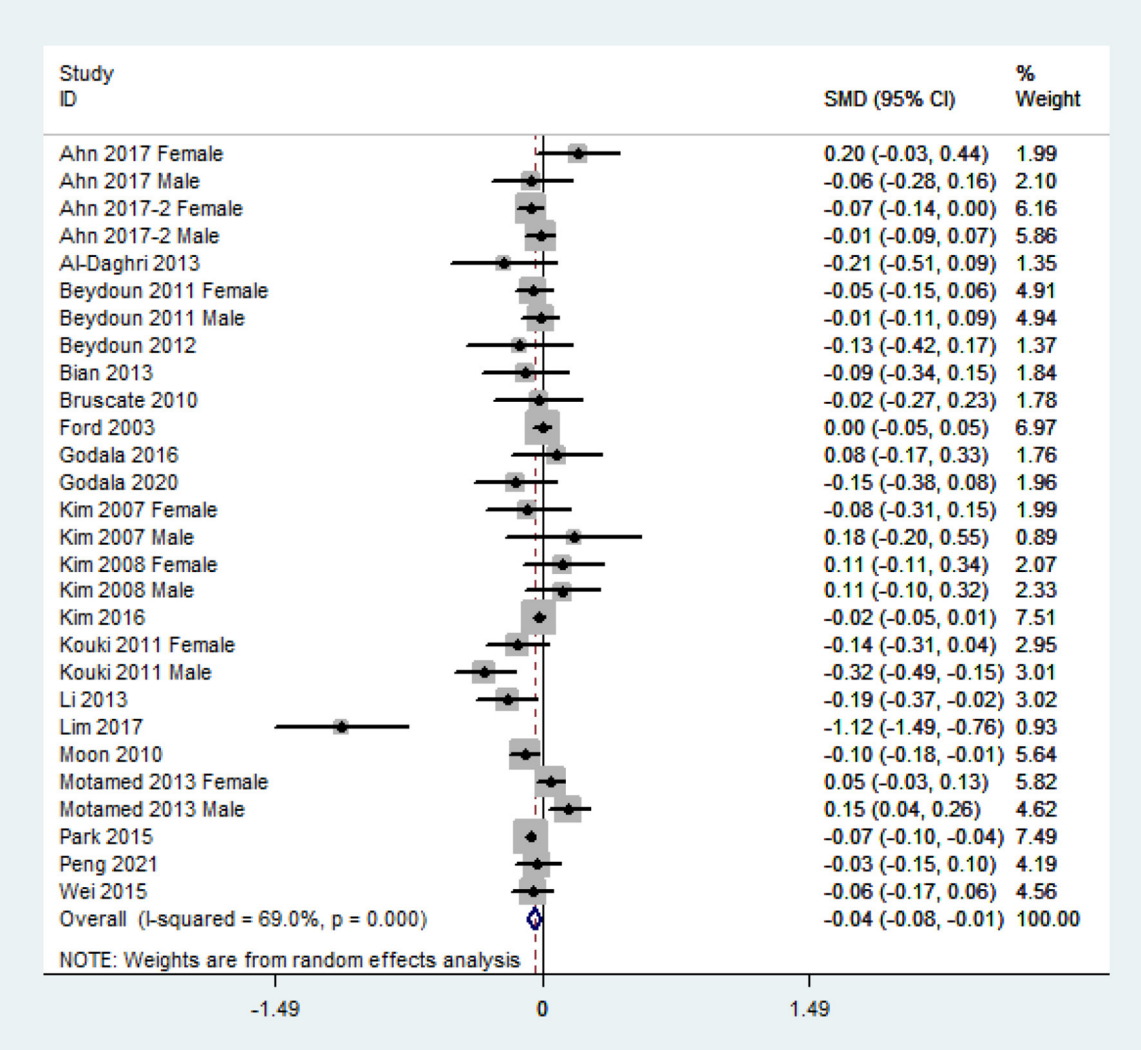
Forest plot of meta-analysis. SMD of dietary vitamin C level for MetS vs. control subjects.

The overall combined SMD showed that the dietary vitamin C level in MetS was lower than that in control subjects (SMD = −0.04, 95% CI: −0.08 to −0.01; *P* = 0.024) (Figure 3). A substantial level of heterogeneity was obtained among the various studies (*P* < 0.001, *I*^2^ = 69.0%). No evidence of publication bias existed according to Begg's rank-correlation test (*P* = 0.314). The results of the subgroup analysis are presented in Table 2. The above findings were confirmed in NCEP ATP III (SMD = −0.06, 95% CI: −0.11 to −0.01; *P* = 0.01), >1,000 sample size (SMD = −0.03, 95%CI: −0.07 to 0.00; *P* = 0.05), 24-h or 3 days recall (SMD = −0.04, 95% CI: −0.08 to 0.00; *P* = 0.05), and high-quality (SMD = −0.04, 95% CI: −0.07 to −0.01; *P* = 0.02) studies.

**Table 2:** Subgroup analysis for standard mean difference (SMD) of dietary vitamin C level in MetS vs. control subjects.

**Stratification**	**Number of studies**	**Pooled SMD**	**95% CI**	***P*-value**	**Heterogeneity**
All studies	21	−0.04	−0.08, −0.01	*P =* 0.02	*P < * 0.001; *I*^2^ = 69%
**Diagnostic criteria of MetS**					
NCEP ATP III	13	−0.06	−0.11, −0.01	*P =* 0.01	*P < * 0.001; *I*^2^ = 72%
Other	8	−0.01	−0.03, 0.02	*P =* 0.65	*P =* 0.07; *I*^2^ = 45%
**Geographical region**					
Asia	14	−0.03	−0.08, 0.01	*P =* 0.16	*P < * 0.001; *I*^2^ = 74%
Non-Asia	7	−0.07	−0.14, 0.00	*P =* 0.06	*P =* 0.04; *I*^2^ = 51%
**Sample size**					
<1,000	11	−0.07	−0.19, 0.05	*P =* 0.23	*P < * 0.001; *I*^2^ = 73%
>1,000	10	−0.03	−0.07, 0.00	*P =* 0.05	*P < * 0.001; *I*^2^ = 66%
**Exposure assessment**					
FFQ	3	0.00	−0.04, 0.04	*P =* 0.95	*P =* 0.40; *I*^2^ = 0%
24 h or 3 days recall	17	−0.04	−0.08, 0.00	*P =* 0.05	*P < * 0.001; *I*^2^ = 69%
**Study quality**					
High-quality	16	−0.04	−0.07, −0.01	*P =* 0.02	*P < * 0.001; *I*^2^ = 57%
Low-quality	5	−0.11	−0.36, 0.13	*P =* 0.38	*P < * 0.001; *I*^2^ = 87%

### RR of MetS for the Highest vs. Lowest Circulating Vitamin C Category

**Figure 4:**
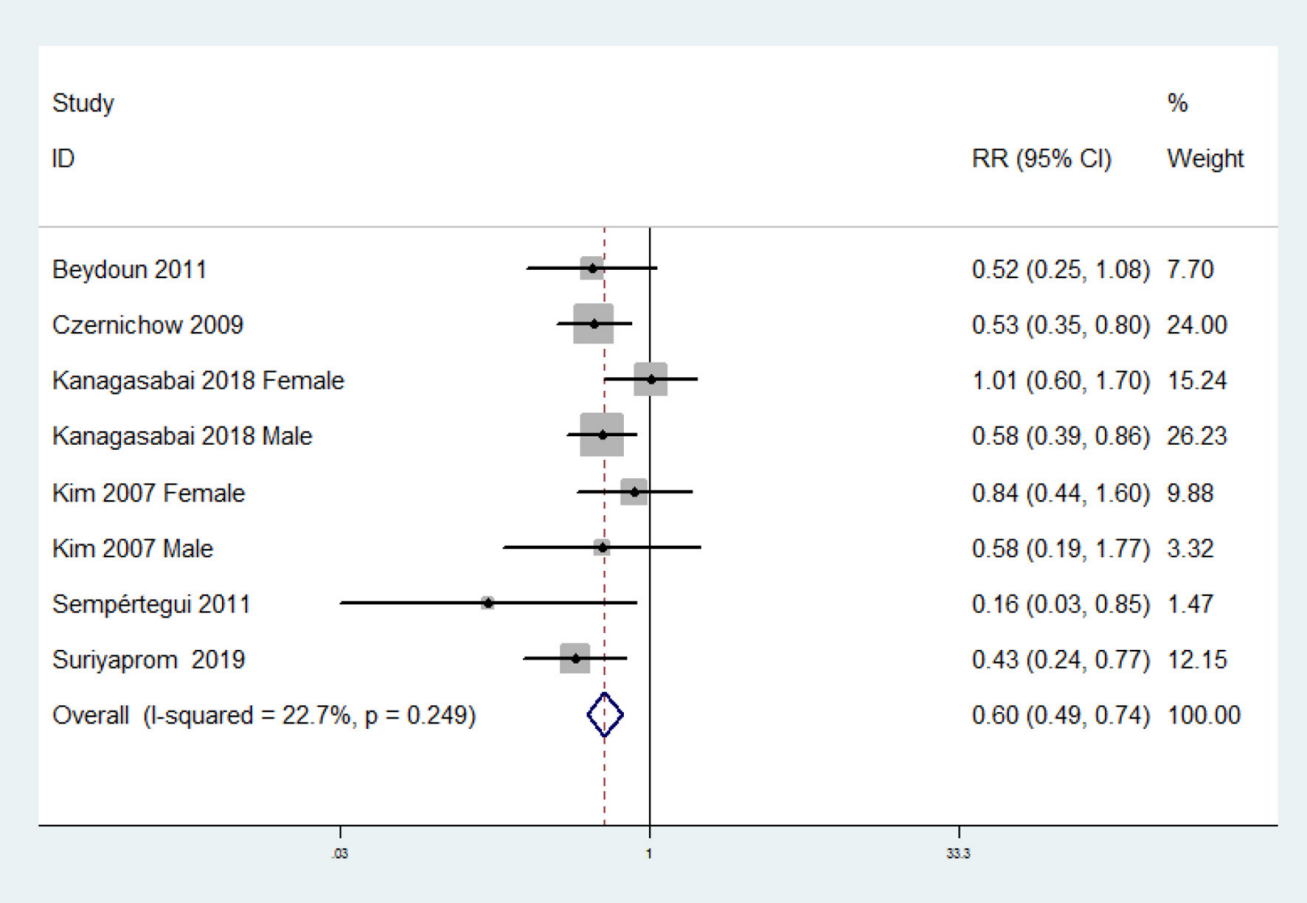
Forest plot of meta-analysis. Overall multi-variable adjusted RR of MetS for the highest vs. lowest category of circulating vitamin C level.

The overall multivariable-adjusted RR demonstrated that the circulating vitamin C level was negatively associated with MetS (RR = 0.60, 95% CI: 0.49 to 0.74; *P* < 0.001) (Figure 4). No substantial level of heterogeneity was obtained among various studies (*P* = 0.249, *I*^2^ = 22.7%). No evidence of publication bias existed according to Begg's rank-correlation test (*P* = 0.536). The results of subgroup analysis were presented in Table 3. The above findings were confirmed in NCEP ATP III (RR = 0.55, 95% CI: 0.42 to 0.72; *P* < 0.001) studies.

**Table 3:** Subgroup analysis of MetS for the highest vs. lowest circulating vitamin C level category.

**Stratification**	**Number of studies**	**Pooled RR**	**95% CI**	***ps*-value**	**Heterogeneity**
All studies	6	0.60	0.49, 0.74	*P < * 0.001	*P =* 0.25; *I*^2^ = 23%
**Study design**					
Crosssectional	5	0.63	0.50, 0.79	*P < * 0.001	*P =* 0.20; *I*^2^ = 30%
Cohort	1	0.53	0.35, 0.80	/	/
**Diagnostic criteria of MetS**					
NCEP ATP III	4	0.55	0.42, 0.72	*P < * 0.001	*P =* 0.66; *I*^2^ = 0%
Other	2	0.63	0.33, 1.19	*P =* 0.15	*P =* 0.06; *I*^2^ = 65%
**Geographical region**					
Asia	2	0.58	0.39, 0.87	*P =* 0.008	*P =* 0.32; *I*^2^ = 12%
Non-Asia	4	0.61	0.48, 0.77	*P < * 0.001	*P =* 0.15; *I*^2^ = 41%
**Sample size**					
<1,000	3	0.54	0.37, 0.80	*P =* 0.002	*P =* 0.22; *I*^2^ = 32%
>1,000	3	0.62	0.49, 0.79	*P =* 0.001	*P =* 0.24; *I*^2^ = 29%
**Study quality**					
High-quality	5	0.63	0.50, 0.79	*P < * 0.001	*P =* 0.20; *I*^2^ = 30%
Low-quality	1	0.53	0.35, 0.80	/	/
**Adjustment of BMI**					
Adjusted	3	0.66	0.51, 0.84	*P =* 0.001	*P =* 0.23; *I*^2^ = 29%
Unadjusted	3	0.50	0.35, 0.71	*P* = 0.001	*P =* 0.39; *I*^2^ = 0%
**Adjustment of physical activity**					
Adjusted	2	0.60	0.43, 0.84	*P =* 0.003	*P =* 0.50; *I*^2^ = 0%
Unadjusted	4	0.60	0.46, 0.77	*P < * 0.001	*P =* 0.10; *I*^2^ = 48%

### SMD of the Circulating Vitamin C Level for MetS vs. Control Subjects

**Figure 5:**
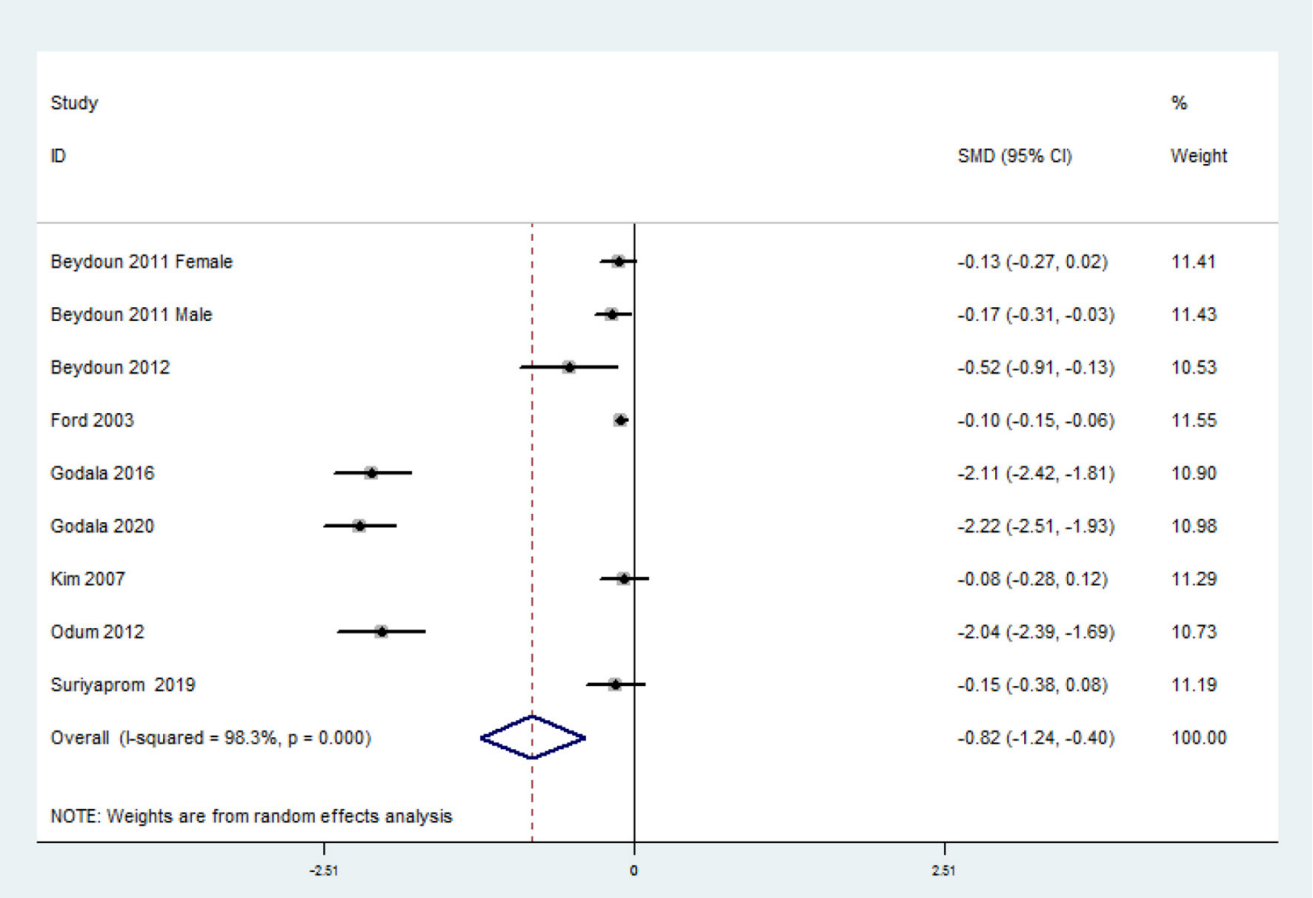
Forest plot of meta-analysis. SMD of circulating vitamin C level for MetS vs. control subjects.

The overall combined SMD showed that the circulating vitamin C level in MetS was lower than that in control subjects (SMD = −0.82, 95% CI: −1.24 to −0.40; *P* < 0.001) (Figure 5). A substantial level of heterogeneity was obtained among the various studies (*P* < 0.001, *I*^2^ = 98.3%). No evidence of publication bias existed according to Begg's rank-correlation test (*P* = 0.076). The results of subgroup analysis are presented in Table 4.

**Table 4:** Subgroup analysis for SMD of circulating vitamin C level in MetS vs. control subjects.

**Stratification**	**Number of studies**	**Pooled SMD**	**95% CI**	***p*-value**	**Heterogeneity**
All studies	8	−0.82	−1.24, −0.40	*P < * 0.001	*P < * 0.001; *I*^2^ = 98%
Diagnostic criteria of MetSNCEP ATP III	5	−0.40	−0.68, −0.11	*P =* 0.006	*P < * 0.001; *I*^2^ = 96%
Other	3	−1.62	−2.59, −0.66	*P =* 0.001	*P < * 0.001; *I*^2^ = 96%
**Geographical region**					
US	3	−0.12	−0.16, −0.07	*P < * 0.001	*P =* 0.18; *I*^2^ = 39%
Non-US	5	−1.31	−2.31, −0.31	*P =* 0.01	*P < * 0.001; *I*^2^ = 99%
**Sample size**					
<1,000	5	−1.31	−2.31, −0.31	*P =* 0.23	*P < * 0.001; *I*^2^ = 99%
>1,000	3	−0.12	−0.16, −0.07	*P < * 0.001	*P =* 0.18; *I*^2^ = 39%
**Study quality**					
High-quality	6	−0.46	−0.80, −0.13	*P =* 0.007	*P < * 0.001; *I*^2^ = 97%
Low-quality	2	−2.08	−2.31, −1.84	*P < * 0.001	*P =* 0.75; *I*^2^ = 0%

## Discussion

In the present meta-analysis, a total of 28 observational studies were identified, and the pooled analysis showed that both the dietary and the circulating vitamin C level were inversely associated with MetS.

Since oxidative stress and inflammation play a significant role in the pathophysiology of MetS (42), the underlying mechanism behind the negative association between vitamin C and MetS can be listed as follows: on the one hand, vitamin C serves as a strong antioxidant, which prevents other compounds from being oxidized (42). Vitamin C can donate electrons, scavenge harmful free radicals, and leave the ascorbyl radical (relatively stable and unreactive) (43, 44). Indeed, antioxidants could lower blood pressure by oxidation of cGMP-dependent protein kinase (45). Several meta-analysis studies have also indicated a potential beneficial effect of vitamins antioxidant on type 2 diabetes (46, 47). A higher intake of antioxidant vitamins and serum total antioxidant status from antioxidant supplementation were associated with a decreased waist circumference and low-density lipoprotein to high-density lipoprotein–cholesterol ratio (40). On the other hand, the neutrophil is an important regulatory marker for acute and chronic inflammation. The hyperplasia and hypertrophy of adipose tissue, the main source of various inflammatory mediators, is closely associated with the MetS-associated inflammation (48). The neutrophil chemotaxis to adipose tissue (with subsequent phagocytosis of microbes and clearance by macrophages) could therefore reduce inflammation. Indeed, vitamin C has the potential to improve neutrophil chemotaxis in both human and animal studies (49–51). In addition, patients with impaired neutrophil bacterial phagocytosis can be significantly improved by vitamin C supplementation (long-lasting clinical improvement) (52). Importantly, vitamin C-deficient neutrophils were not phagocytosed by macrophages *in vitro* and persisted at inflammatory loci *in vivo* (53). Taken together, vitamin C is able to alleviate the inflammatory response by influencing neutrophil chemotaxis in response to inflammatory mediators, enhancing phagocytosis of microbes by neutrophils and neutrophil clearance by macrophages (52).

The effect of vitamin C on MetS has been extensively investigated in experimental animal and clinical studies. Bilbis et al. found that 4-week supplementation of vitamin C could decrease the weight gain, blood pressure, glucose, insulin, insulin resistance, total cholesterol, triglycerides, low-density lipoprotein cholesterol, and very low-density lipoprotein cholesterol in a salt-loaded animal model (54), indicating a strong biological effect of vitamin C on the circulating profile in MetS. Moreover, unlike animals, humans are dependent upon dietary vitamin C due to mutations in l-gulono-γ-lactone oxidase (Gulo, the final enzyme for vitamin C biosynthesis). The induction of vitamin C deficiency in Gulo-/- mice was associated with a phenotype characterized by insulin resistance, weight gain, dyslipidemia, and hepatic steatosis (7). Indeed, this animal model was humanized and necessarily reflected the issue. Importantly, Abd indicated that the combination of vitamin C and l-methionine had the most beneficial effect on hyperglycemia, dyslipidemia, abnormal coagulation indices, and oxidative stress in the alloxan-induced diabetes model. Asynergistic biological effects may exist between vitamin C and others, which indicated that the combination therapy strategy for vitamin C is promising (55). With regard to the clinical evidence, Farag et al. found that vitamin C supplementation could result in a significant reduction in BMI, and the combination of physical activities [exercise alone was reported to be beneficial to MetS (56, 57)] and vitamin C supplements may further improve systolic blood pressure and serum levels of total cholesterol in MetS patients (58, 59). Taken together, the current experimental and clinical evidence strongly supports the potential beneficial effect of vitamin C on MetS. Moreover, the vitamin C combination therapy (with l-methionine or exercise) seems to be a promising strategy for the management of MetS, which might be recommended clinically.

Interestingly, the inverse relationship between dietary vitamin C level and MetS was only obtained in crosssectional studies. However, the number of cohort studies is rather small (only two), which may inevitably reduce the reliability. Although vitamin C may be beneficial to the components or complications of MetS (e.g., decreased blood pressure, glucose, insulin, insulin resistance, etc.), it is not necessarily a reflection of MetS prevention. One cohort study combined vitamin C and B_1_ as a whole, and the effect of vitamin B_1_ could not be specified (27). Interestingly, the inconsistent result with regard to diagnostic criteria of MetS and exposure assessment was acquired. It was speculated that NCEP ATP III criteria and recall method seems to be more precise and suitable for vitamin C evaluation. With regard to the SMD analysis for the dietary vitamin C levels, the findings disappeared in small sample-sized (<1,000) studies. Indeed, large sample-sized studies may be more reliable to address the issues. Importantly, no study has specified the dietary season variation, and our results might be influenced by the seasonal variability of vitamin C levels. Taken together, more well-designed prospective cohort studies are still needed.

Our study has several strengthens. To begin with, this is the first meta-analysis of observational studies on the associations of dietary and circulating vitamin C levels with MetS. In addition, the included studies are analyzed based on the adjusted results and large samples. Finally, our findings are consistent with the current corresponding experimental and clinical studies. Above all, our study might provide helpful information to better consider the effect of vitamin C on MetS.

On the other hand, we should also acknowledge the limitations of this study. (1) The substantial level of heterogeneity might have distorted the reliability of our results. (2) Due to the limited relevant literature, only three prospective cohort studies were identified totally (causal relationships could not be obtained). (3) The classification of exposure may vary greatly among individuals. (4) The selection of adjusted factors and definition of MetS was not uniform. (5) One included study has combined the effect estimates for vitamin C and vitamin B_1_ as a whole (27), some issues could not be addressed. (6) The seasonal variation in vitamin C level cannot be considered in this study. These limitations may weaken the significance of our study.

## Conclusions

Current evidence suggests that both dietary and circulating vitamin C levels are inversely associated with MetS. However, due to the limitation of the available evidence, more well-designed prospective studies are still needed.

## Data Availability Statement

Publicly available datasets were analyzed in this study. This data can be found at: PubMed, Web of Science, Embase.

## Author Contributions

YZ, HG, and JD conceived the idea, performed the statistical analysis, and drafted this meta-analysis. YZ and JD selected retrieved relevant papers. QL and YL assessed each study. YZ was the guarantors of the overall content. All authors revised and approved the final manuscript.

## Funding

This study was supported by the National Natural Science Foundation of China (82102581), the National Postdoctoral Science Foundation of China (2021M693562), the Provincial Outstanding Postdoctoral Innovative Talents Program of Hunan (2021RC2020), the Young Investigator Grant of Xiangya Hospital, Central South University (2020Q14), and the FuQing Postdoc Program of Xiangya Hospital, Central South University (176).

## Conflict of Interest

The authors declare that the research was conducted in the absence of any commercial or financial relationships that could be construed as a potential conflict of interest.

## Publisher's Note

All claims expressed in this article are solely those of the authors and do not necessarily represent those of their affiliated organizations, or those of the publisher, the editors and the reviewers. Any product that may be evaluated in this article, or claim that may be made by its manufacturer, is not guaranteed or endorsed by the publisher.
