# Supplementary material for: Vitamin C and Metabolic Syndrome: A Meta-Analysis of Observational Studies
Source: Front Nutr. 2021 Oct 8;8:728880. doi: 10.3389/fnut.2021.728880 (PMC8531097; doi:10.3389/fnut.2021.728880)
Supplement: Supplementary file 1 [file Table_1.docx]

Supplementary table 1 Characteristics of the individual studies included in this meta-analysis

| First author  year of  publication | Location | Age  years | Gender | Sample Size | Study design | Adjustments | Exposure | Category of exposure | Effect Estimates  (RR or SMD) | Diagnostic criteria of MetS | NOS |
| --- | --- | --- | --- | --- | --- | --- | --- | --- | --- | --- | --- |
| Ford 2003 [8] | US | >20 | Both | 8808 | Cross-sectional | NA | FFQ | Control subjects  MetS subjects  Control subjects  MetS subjects | Dietary vitamin C  106.3 (100.8, 111.8)  106.4 (96.0, 116.8)  Circulating vitamin C  42.9 (41.3, 44.6)  36.4 (34.2, 38.6) | NCEP-ATP III | 8 |
| Kim 2007 [9] | Korea | >60 | Both | 404 | Cross-sectional | Age, BMI, energy intake, smoking status, alcohol, physical activity, vitamin, and mineral supplements | 24 hours recall | Male  Control subjects  MetS subjects  Female  Control subjects  MetS subjects  Dietary vitamin C  Male  Quartiles 1  Quartiles 2  Quartiles 3  Quartiles 4  Female  Quartiles 1  Quartiles 2  Quartiles 3  Quartiles 4  Control subjects  MetS subjects  Circulating vitamin C  Male  Tertiles 1  Tertiles 2  Tertiles 3  Female  Tertiles 1  Tertiles 2  Tertiles 3 | Dietary vitamin C  124.2 (101.1, 147.3)  144.4 (102.0, 186.8)  Dietary vitamin C  107.8 (94.6, 121.0)  101.4 (88.2, 114.6)  RR  1.0  1.2 (0.4, 3.6)  1.5 (0.54, 4.5)  0.58 (0.19, 1.8)  RR  1.0  1.1 (0.60, 2.3)  1.0 (0.53, 1.98)  0.84 (0.44, 1.63)  Circulating vitamin C  514.7 (454.2, 575.2)  481.7 (431.8, 531.6)  RR  1.0  0.66 (0.13, 3.20)  0.45 (0.10, 2.40)  RR  1.0  1.40 (0.59, 3.30)  1.40 (0.62, 3.50) | NCEP-ATP III | 7 |
| Kim 2008 [10] | Korea | Middle-aged | Both | 688 | Cross-sectional | NA | FFQ | Male  Control subjects  MetS subjects  Female  Control subjects  MetS subjects | Dietary vitamin C  56.6 (52.8, 60.4)  59.7 (55.0, 64.4)  Dietary vitamin C  70.0 (64.8, 75.2)  73.9 (69.0, 78.8) | NCEP-ATP III | 6 |
| Czernichow 2009 [29] | France | 49 | Both | 5520 | Cohort | Age, sex, intervention group, educational level, smoking status, physical activity and alcohol consumption | NA | Circulating vitamin C  Tertiles 1  Tertiles 2  Tertiles 3 | RR  1.0  0.76 (0.53, 1.09)  0.53 (0.35, 0.80) | NCEP-ATP III | 5 |
| Moon 2010 [11] | Korea | 40-64 | Both | 2382 | Cross-sectional | NA | 24 hours recall | Control subjects  MetS subjects | Dietary vitamin C  111.8 (107.4, 116.2)  103.3 (97.6, 109.0) | NCEP-ATP III | 7 |
| Bruscate 2010 [34] | Brazil | 69.3 | Female | 284 | Cross-sectional | Age, smoking, education, physical activity and dietary fiber | 24 hours recall | Control subjects  MetS subjects  Dietary vitamin C  Quartiles 1  Quartiles 2  Quartiles 3  Quartiles 4 | Dietary vitamin C  135.0 (121.9, 148.1)  133.0 (112.8, 153.2)  RR  1.0  0.69 (0.33, 1.46)  0.55 (0.26, 1.18)  0.70 (0.33, 1.47) | IDF | 6 |
| Beydoun 2011 [12] | US | 20-85 | Both | 3202 | Cross-sectional | Age, sex, race/ethnicity, marital status, educational level, PIR, smoking status, total energy intake, alcohol, caffeine, b-carotene, vitamin C, vitamin E, and dietary supplement use, serum levels of folate, tHcy, vitamin B-12, 25(OH)D, total cholesterol, and TG | 24 hours recall | Male  Control subjects  MetS subjects  Female  Control subjects  MetS subjects  Male  Control subjects  MetS subjects  Female  Control subjects  MetS subjects  Circulating vitamin C  Quartiles 1  Quartiles 2  Quartiles 3  Quartiles 4 | Dietary vitamin C  96.5 (90.0, 103.0)  95.3 (83.7, 106.9)  Dietary vitamin C  85.4 (79.9, 90.9)  81.2 (73.5, 88.9)  Circulating vitamin C  50.9 (47.3, 54.5)  43.7 (39.7, 47.7)  Circulating vitamin C  57.7 (54.5, 60.9)  52.1 (45.2, 59.0)  RR  1.0  0.98 (0.62, 1.53)  0.52 (0.28, 0.98)  0.52 (0.25, 1.10) | NCEP-ATP III | 8 |
| Sempértegui 2011 [28] | Ecuador | >65 | Both | 352 | Cross-sectional | Age and sex | NA | Circulating vitamin C  < 0.2 mg/dL  > 0.2 mg/dL | RR  1.0  0.16 (0.03, 0.81) | IDF | 7 |
| Kouki 2011 [13] | Finland | 57-78 | Both | 1334 | Cross-sectional | Age, alcohol consumption, smoking, education and VO_2_ max | 4-day food record | Male  Control subjects  MetS subjects  Female  Control subjects  MetS subjects  Dietary vitamin C  Male  < 10mg/d  > 10mg/d  Female  < 10mg/d  > 10mg/d | Dietary vitamin C 112.0 (105.7, 118.3)  90.0 (80.6, 99.4)  Dietary vitamin C 115.0 (110.0, 120.1)  107.0 (98.3, 115.7)  RR  1.0  0.97 (0.94, 1.01)  RR  1.0  0.99 (0.96, 1.02) | NCEP-ATP III | 7 |
| Beydoun 2012 [14] | US | 12-19 | Both | 1339 | Cross-sectional | NA | 24 hours recall | Control subjects  MetS subjects  Control subjects  MetS subjects | Dietary vitamin C  83.8 (76.5, 91.1)  67.3 (41.7, 92.9)  Circulating vitamin C  54.9 (51.8, 58.0)  32.6 (22.5, 42.7) | IDF | 7 |
| de Oliveira Otto 2012 [15] | US | 45-84 | Both | 3828 | Cohort | Energy intake, age, sex, race-ethnicity, education, study center, alcohol intake, physical activity, BMI, fiber intake, cigarette smoking, dietary supplement use, the ratio of polyunsaturated fat intake and saturated fat intake, Mg, Zn, heme iron, nonheme iron, and antioxidant intake | FFQ | Dietary vitamin C  Quintiles 1  Quintiles 2  Quintiles 3  Quintiles 4  Quintiles 5 | RR  1.0  1.07 (0.85, 1.34)  1.04 (0.82, 1.32)  1.01 (0.79, 1.29)  1.18 (0.90, 1.54) | AHA | 8 |
| Odum 2012 [30] | Nigeria | 50 | Both | 192 | Case-control | NA | NA | Control subjects  MetS subjects | Circulating vitamin C  43.9 (42.5, 45.4)  29.1 (27.6, 30.5) | NCEP-ATP III | 6 |
| Al-Daghri 2013 [16] | Saudi Arabia | 19-60 | Both | 185 | Cross-sectional | Age, BMI and physical activity | 24 hours recall | Control subjects  MetS subjects  Dietary vitamin C  Quartiles 1  Quartiles 2  Quartiles 3  Quartiles 4 | Dietary vitamin C  61.2 (49.9, 72.5)  50.2 (42.0, 58.4)  RR  1.0  0.44 (0.21, 0.66)  0.24 (0.15, 0.32)  0.24 (0.09, 0.49) | IDF | 7 |
| Motamed 2013 [33] | Iran | 35-65 | Both | 3800 | Cross-sectional | Sex, age, physical activity level, smoking, past medical history, energy intake, and BMI | 24 hours recall | Male  Control subjects  MetS subjects  Female  Control subjects  MetS subjects  Dietary vitamin C  Quintiles 1  Quintiles 2  Quintiles 3  Quintiles 4  Quintiles 5 | Dietary vitamin C  84.9 (78.5, 90.7)  97.4 (89.7, 105.1)  Dietary vitamin C  80.4 (76.3, 84.6)  84.2 (79.5, 88.9)  RR  1.0  1.01 (0.80, 1.20)  0.89 (0.70, 1.10)  1.02 (0.80, 1.20)  1.08 (0.80, 1.30) | IDF | 8 |
| Bian 2013 [17] | China | 30-70 | Both | 258 | Cross-sectional | NA | 24 hours recall | Control subjects  MetS subjects | Dietary vitamin C  106.0 (98.1, 113.9)  101.9 (94.5, 109.3) | NCEP-ATP III | 9 |
| Li 2013 [18] | China | 18-65 | Both | 550 | Cross-sectional | Age and sex | 3-day food record | Control subjects  MetS subjects  Dietary vitamin C  Quartiles 1  Quartiles 2  Quartiles 3  Quartiles 4 | Dietary vitamin C  62.0 (56.8, 67.2)  53.2 (47.7, 58.7)  RR  1.0  0.77 (0.46,1.30)  0.63 (0.37-1.06)  0.63 (0.37-1.06) | NCEP-ATP III | 7 |
| Park 2015 [19] | Korea | >20 | Both | 27656 | Cross-sectional | NA | 24 hours recall | Control subjects  MetS subjects | Dietary vitamin C  80.1 (78.7, 81.5)  73.4 (71.0, 75.8) | NCEP-ATP III | 8 |
| Wei 2015 [20] | China | 18-84 | Both | 2069 | Cross-sectional | Age, sex, cigarette smoking, alcohol, drinking, nutritional supplementary, activity level, dietary energy intake, fiber intake and protein intake. | FFQ | Control subjects  MetS subjects  Dietary vitamin C  Quartiles 1  Quartiles 2  Quartiles 3  Quartiles 4 | Dietary vitamin C  108.8 (104.8, 112.8)  104.2 (95.9, 112.5)  RR  1.0  0.65 (0.47, 0.90)  0.79 (0.57, 1.10)  0.64 (0.43, 0.94) | AHA | 7 |
| Kim 2016 [22] | Korea | >20 | Both | 22671 | Cross-sectional | Age, sex, BMI, smoking, education level, household income, energy intake, total fat intake, dietary fiber intake and alcohol consumption | 24 hours recall | Control subjects  MetS subjects  Dietary vitamin C  <85 mg/day  ≥85 mg/day | Dietary vitamin C  111.8 (109.4, 114.2)  109.4 (105.7, 113.1)  RR  1.0  0.89 (0.80, 0.99) | JIS | 8 |
| Godala 2016 [21] | Poland | 30-65 | Both | 182 | Cross-sectional | NA | 3-day food record | Control subjects  MetS subjects  Control subjects  MetS subjects | Dietary vitamin C  93.3 (66.7, 120.0)  104.0 (83.6, 124.3)  Circulating vitamin C  58.4 (54.7, 62.2)  31.2 (29.8, 32.5) | IDF | 6 |
| Lim 2017 [24] | Korea | Middle-aged | Both | 143 | Cross-sectional | Not mentioned | 3-day food record | Control subjects  MetS subjects  Dietary vitamin C  Low-consumer  High-consumer | Dietary vitamin C  92.7 (88.3, 97.1)  63.1 (53.3, 72.9)  RR  1.0  0.89 (0.84, 0.94) | NCEP-ATP III | 6 |
| Ahn 2017 [23] | Korea | 30-60 | Both | 614 | Cross-sectional | Age, smoking status, alcohol consumption and physical activity | 3-day food record | Male  Control subjects  MetS subjects  Female  Control subjects  MetS subjects  Dietary vitamin C  Male  Tertile 1  Tertile 2  Tertile 3  Female  Tertile 1  Tertile 2  Tertile 3 | Dietary vitamin C  41.1 (36.4, 45.8)  39.4 (35.0, 43.8)  Dietary vitamin C  52.4 (47.0, 57.8)  60.4 (52.7, 68.1)  RR  1.0  1.12 (0.64, 1.97)  0.87 (0.49, 1.53)  RR  1.0  0.76 (0.42, 1.36)  1.24 (0.69, 2.21) | NCEP-ATP III | 7 |
| Ahn 2017-2 [35] | Korea | 20-65 | Both | 10286 | Cross-sectional | NA | 24 hours recall | Male  Control subjects  MetS subjects  Female  Control subjects  MetS subjects | Dietary vitamin C  106.7 (102.0, 115.5)  105.1 (91.5, 118.7)  Dietary vitamin C  112.1 (107.0, 117.3)  99.6 (89.0, 110.1) | NCEP-ATP III | 8 |
| Kanagasabai 2018 [32] | Canada | >20 | Both | 2049 | Cross-sectional | Age, ethnicity, education, income, smoking, alcohol intake, recreational PA, and BMI | NA | Circulating vitamin C  Male  Lower  Normal  Female  Lower  Normal | RR  1.0  0.58 (0.39, 0.98)  RR  1.0  0.52 (0.28, 0.98) | JIS | 7 |
| Ahn 2019 [25] | Korea | 19-64 | Both | 10351 | Cross-sectional | Age, BMI, alcohol consumption, smoking, physical activity, household income, education level and energy intake | 24 hours recall | Dietary vitamin C  Male  Tertile 1  Tertile 2  Tertile 3  Female  Tertile 1  Tertile 2  Tertile 3 | RR  1.0  0.88 (0.70, 1.09)  0.75 (0.58, 0.95)  RR  1.0  1.24 (0.96, 1.61)  0.81 (0.62, 1.06) | NCEP-ATP III | 7 |
| Suriyaprom 2019 [31] | Thailand | 30-59 | Both | 300 | Case-control | Age, gender, and BMI | NA | Control subjects  MetS subjects  Circulating vitamin C  < 5mg/L  > 5mg/L | Circulating vitamin C  6.70 (6.00, 7.50)  5.90 (4.80, 6.70)  RR  1.0  0.43 (0.24, 0.75) | NCEP-ATP III | 7 |
| Godala 2020 [26] | Poland | 57 | Both | 332 | Cross-sectional | NA | 24 hours recall | Control subjects  MetS subjects  Control subjects  MetS subjects | Dietary vitamin C  127.9 (108.0, 147.8)  113.1 (100.3, 125.8)  Circulating vitamin C  64.2 (61.4, 67.0)  35.3 (33.7, 36.9) | IDF | 7 |
| Peng 2021 [27] | China | >99 | Both | 992 | Cohort | Aex, marital status, physical activity, smoking status, alcohol intake, family history of chronic diseases and daily total energy intake | 24 hours recall | Control subjects  MetS subjects  Dietary vitamin C  Quartiles 1  Quartiles 2  Quartiles 3  Quartiles 4 | Dietary vitamin C  88.5 (56.0, 121.0)  79.7 (53.1, 106.3)  RR  1.0  1.28 (0.79, 2.02)  0.81 (0.47, 1.37)  0.91 (0.53, 1.84) | NCEP-ATP III | 6 |
